# Thermal ablation for papillary thyroid microcarcinoma in the isthmus: a systematic review and meta-analysis

**DOI:** 10.1530/EC-25-0268

**Published:** 2025-07-24

**Authors:** Yuqi Hao, Kun Liao, Ling Zeng, Lili Feng

**Affiliations:** ^1^ Jiangxi Medical College, Nanchang University, Nanchang, China; ^2^ Jiangxi Provincial People’s Hospital, The First Affiliated Hospital of Nanchang Medical College, Nanchang, China; ^3^ Jiangxi Maternal and Child Health Hospital, Nanchang, China

**Keywords:** thyroid cancer, papillary thyroid microcarcinoma, isthmus, thermal ablation, systematic review

## Abstract

**Background:**

The treatment strategy for isthmic papillary thyroid microcarcinoma (PTMC) remains controversial. Although preliminary studies have confirmed the safety and efficacy of thermal ablation (TA), systematic reviews focusing on isthmic PTMC are lacking. This study is the first meta-analysis to comprehensively evaluate the clinical value of TA for isthmic PTMC.

**Methods:**

A comprehensive search was conducted across PubMed, EMBASE, Cochrane Library, and Web of Science databases from their establishment dates through February 21, 2025, aiming to locate relevant studies investigating TA-treated isthmic PTMC cases. Following PRISMA standards, two investigators independently carried out three essential procedures: screening eligible literature, collecting pertinent data, and assessing study quality through established evaluation criteria.

**Results:**

Five studies involving 364 patients were included. Primary outcomes showed that the volume reduction rate at 12 months post-TA was 98.27% (95% CI: 96.95–99.58), with 90.84% (95% CI: 70.08–100.00) achieving complete tumor disappearance. During follow-up, the local recurrence rate was 0.38% (3/364; 95% CI: 0.00–1.65), and the lymph node metastasis rate was 0.01% (1/364; 95% CI: 0.00–0.77). Regarding safety, the major complication rate was 0.06% (2/364; 95% CI: 0.00–0.09%), both transient voice hoarseness, with no severe complications or distant metastasis reported.

**Conclusion:**

TA demonstrates excellent tumor control and safety for isthmic PTMC. However, current evidence is limited by retrospective study designs (80%) and short follow-up periods (mean <5 years). Future large-scale, multicenter prospective studies with longer follow-up are needed.

**Registration information:**

Registration number: INPLASY202520104; date: February 23, 2025.

## Introduction

Thyroid carcinoma represents the predominant malignancy within the endocrine system (1). Among thyroid cancers, papillary thyroid carcinoma (PTC) accounts for approximately 85% of histopathological classifications (1). Epidemiological data indicate that 2.5–9.2% of PTC cases originate from the thyroid isthmus (2). While clinical guidelines have established consensus on managing lateral lobe PTC (3), therapeutic strategies for isthmic tumors remain contentious.

Due to its indolent nature, active surveillance (AS) is recommended as the first-line management for PTMC (4). However, patient anxiety often leads to preference for surgical intervention. The isthmus, anatomically adjacent to the anterior cervical muscles and posteriorly adherent to the second to fourth tracheal cartilage rings, has a unique relationship with the pretracheal fascia. Some researchers suggest that this proximity contributes to greater invasiveness in isthmic PTC compared to lobar PTC, prompting most scholars to advocate surgical resection (5). However, surgical intervention may lead to permanent hypoparathyroidism, recurrent laryngeal nerve damage, and scar formation – complications that can substantially impair a patients’ quality of life (6, 7).

TA, a minimally invasive technique, has shown promise in PTMC treatment. While extensive studies validate its safety and efficacy for lobar tumors (8, 9, 10), few focus on isthmic lesions. Therefore, this PRISMA-compliant systematic review and meta-analysis aims to comprehensively assess the safety and efficacy of TA for isthmic PTMC, providing critical insights for clinical decision-making.

## Materials and methods

This study was conducted in accordance with the Preferred Reporting Items for Systematic Reviews and Meta-Analyses (PRISMA) guidelines for systematic reviews and meta-analyses (11). Before implementation, the study protocol underwent formal registration on the International Platform of Registered Systematic Review and Meta-Analysis Protocols (INPLASY).

### Literature search

We conducted a comprehensive search across four major biomedical databases (PubMed, EMBASE, Web of Science, and Cochrane Library), encompassing all available records from their establishment dates through February 21, 2025. The search strategy in PubMed is shown in Table 1.

**Table 1:** Retrieval steps.

ID	Search terms
#1	‘Thyroid cancer, papillary’[mesh]
#2	(Cancer, papillary thyroid[title/abstract]) OR (cancers, papillary thyroid[title/abstract]) OR (papillary thyroid cancer[title/abstract]) OR (papillary thyroid cancers[title/abstract]) OR (thyroid cancers, papillary[title/abstract]) OR (papillary thyroid carcinoma[title/abstract]) OR (thyroid carcinoma, papillary[title/abstract]) OR (carcinoma, papillary thyroid[title/abstract]) OR (carcinomas, papillary thyroid[title/abstract]) OR (papillary thyroid carcinomas[title/abstract]) OR (thyroid carcinomas, papillary[title/abstract]) OR (papillary carcinoma of thyroid[title/abstract]) OR (papillary thyroid microcarcinoma[title/abstract]) OR (thyroid papillary microcarcinoma[title/abstract]) OR (papillary thyroid micro-carcinoma[title/abstract]) OR (thyroid microcarcinoma[title/abstract])
#3	‘Radiofrequency ablation’[mesh]
#4	(Thermal ablation[title/abstract]) OR (microwave ablation[title/abstract]) OR (radiofrequency ablation[title/abstract]) OR (laser ablation[title/abstract])
#5	#1 OR #2
#6	#3 OR #4
#7	#5 AND #6

### Inclusion criteria

The inclusion criteria comprised five key requirements: i) pathological confirmation of PTC diagnosis; ii) maximum tumor diameter ≤10 mm; iii) lesions confined to the thyroid isthmus; iv) no evidence of extrathyroidal extension (ETE), LNM, or distant metastases; and v) ultrasound-guided TA procedures (radiofrequency/microwave/laser ablation, RFA/MWA/LA).

### Exclusion criteria

The exclusion criteria comprised the following categories: i) case series or reports containing less than 20 participants (*n* < 20); ii) review articles, systematic analyses, or meta-analytic studies; iii) letters, conference abstracts, guidelines, or editorials; iv) animal experiments or *in vitro* studies; and v) duplicate publications or studies with overlapping populations.

### Data extraction

Data collection employed standardized templates systematically organized into three domains: i) study characteristics, including the first author, publication year, institutional affiliation, study design (prospective or retrospective), ablation modality, sample size, sex ratio, mean age, and inclusion/exclusion criteria; ii) technical parameters of ablation, encompassing ablation device, operator experience, power output (W), energy delivery (J), mean ablation time, anesthesia type, application of moving-shot or hydrodissection techniques, ablation margin distance from the tumor edge, and methods for confirming ablation completeness; and iii) ablation outcomes, covering the mean follow-up duration, pre-ablation tumor dimensions (maximum diameter and volume), volume reduction rate at 12 months and final follow-up (calculated as [(initial tumor volume−final tumor volume) × 100]/initial tumor volume), complete tumor disappearance rate, local recurrence rate (defined as tumor regrowth at the ablation site or new tumors in other thyroid regions), lymph node metastasis and distant metastasis rates, and major complications: esophageal/tracheal injury, severe hemorrhage, skin burns, transient/permanent voice changes, hypothyroidism, or hypoparathyroidism. Minor postoperative adverse events were categorized as side effects.

### Quality assessment

Two investigators independently conducted quality appraisal of selected studies using the Newcastle–Ottawa Scale (NOS) (12), focusing on three critical dimensions: i) appropriateness of cohort selection criteria, ii) methodological comparability across study groups, and iii) the validity of outcome evaluation procedures, employing a 9-point scoring system for comprehensive assessment.

### Data synthesis and analyses

The primary outcome variables included pooled proportions of the volume reduction rate at 12 months, complete tumor disappearance rate, tumor recurrence, lymph node metastasis, and major complications. Inter-study heterogeneity was evaluated using the Cochran’s Q test with a prespecified significance level of *P* < 0.1. Outcomes demonstrating *P* ≥ 0.1 were considered homogeneous, whereas *P* < 0.1 indicated significant heterogeneity. To address variability, random-effects models were applied for analyses with substantial heterogeneity (*I*^2^ > 50%), and fixed-effects models for those with minimal variability (*I*^2^ ≤ 50%). Graphical representations of effect estimates were produced via forest plots. Statistical computations were performed by investigator Yuqi Hao using the *metan* and *metaprop* packages in Stata 18.0.

## Results

### Literature search

**Figure 1:**
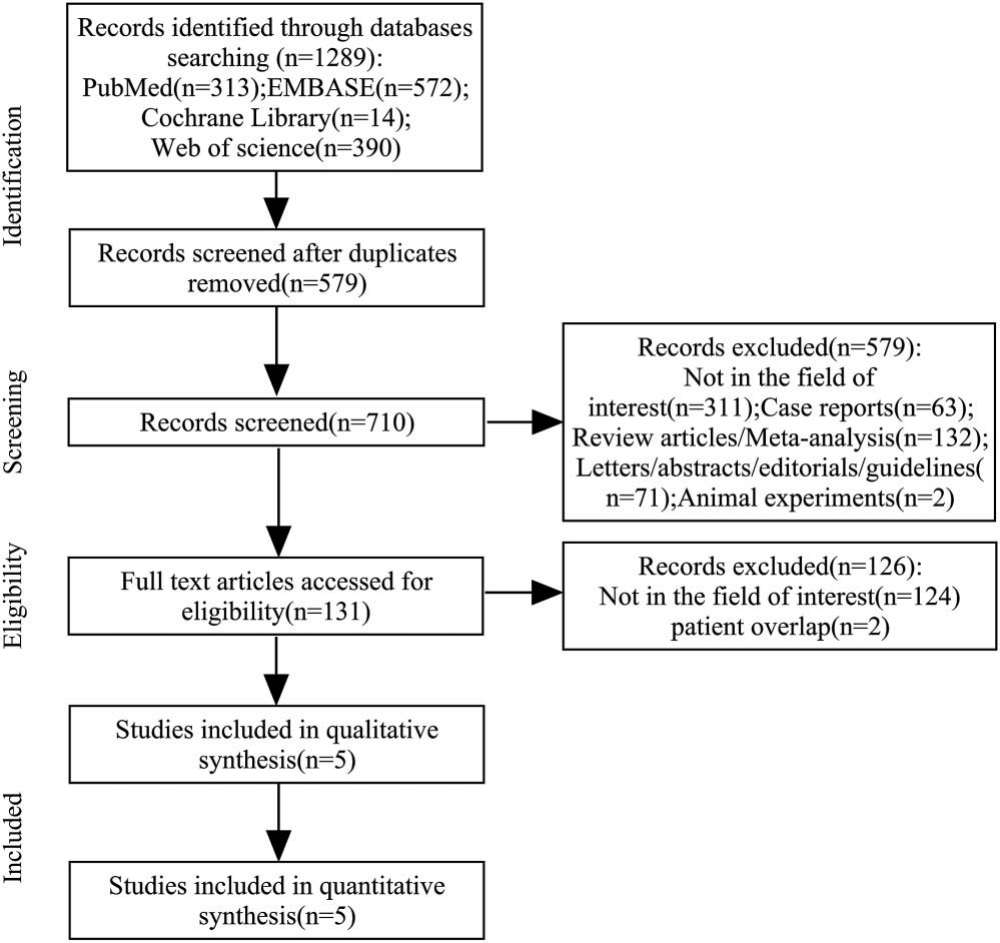
Flowchart of search of articles.

Figure 1 illustrates the study selection workflow. Initial database searches identified 1,289 records across four electronic sources. Following exclusion of 579 duplicate records, 710 unique articles underwent subsequent analysis. Screening of titles and abstracts excluded 579 articles, including 311 irrelevant studies, 63 case reports or case series, 132 review articles or meta-analyses, 71 letters/conference abstracts/guidelines, and two animal studies. Full-text reviews were conducted for the remaining 131 articles, of which 124 were excluded due to irrelevance and three were suspected of overlapping cohorts (the study with the larger sample size was retained) (13, 14, 15). Five articles were ultimately included in the analysis (15, 16, 17, 18, 19).

### Characteristics of the included studies

As shown in Table 2, this analysis incorporated five clinical studies with a cumulative total of 364 patients, including four retrospective (15, 17, 18, 19) studies and one prospective study (16). Regarding ablation modalities, three studies utilized RFA (15, 16, 17), while the remaining two employed MWA (18, 19). The mean age of participants across the studies ranged from 41.07 to 45.05 years, with individual study sample sizes varying between 21 and 135 cases. A notable female predominance was observed in all studies, with a female-to-male ratio ranging from 1.33:1 to 7.43:1. Geographically, all studies were conducted at single-center institutions in China, indicating potential regional selection bias. The inclusion criteria were largely consistent across studies: i) histopathologically confirmed PTC; ii) tumor located in the thyroid isthmus with a maximum diameter ≤10 mm; iii) absence of lymph node metastasis or distant metastasis on imaging; iv) no evidence of extrathyroidal extension; and v) patient refusal of surgery or ineligibility for surgical intervention. One study additionally excluded cases with capsular invasion, and all studies required participants to have no severe coagulation dysfunction or major organ failure.

**Table 2:** Characteristics of the included studies.

First author, year	Affiliation	Study design	Type of TA	No. of patients	Male/female	Mean ± SD age (y)	Inclusion criteria for ablation (summary of inclusion and exclusion criteria)
Wang, 2025 (16)	The 983rd Hospital of Joint Logistic support Force of PLA, China	Prospective	RFA	135	26/109	41.07 ± 13.60	i) Single isthmus PTMC; ii) no LNM/ETE/distant metastasis; iii) ineligibility for or refusal of surgery/active surveillance; iv) no pregnancy; v) follow-up time >24 months; vi) no previous thyroid surgery or thermal ablation; vii) clear imaging quality
Zhou, 2024 (17)	Hangzhou Weja Hospital, China	Retrospective	RFA	59	7/52	43.79 ± 12.04	i) Single isthmus T1aN0M0 PTC; ii) patient refusal of surgery or other treatments; iii) follow-up ≥12 months; iv) no capsule contact/invasion; v) no severe coagulation disorders; vi) no dysfunction/failure of vital organs (heart, liver, or kidney)
Zheng, 2022 (18)	Chinese PLA General Hospital, China	Retrospective	MWA	21	9/12	45.05 ± 9.17	i) Single isthmus PTMC; ii) no LNM/ETE; iii) ineligibility for or refusal of surgery; iv) no clinically apparent multifocality; v) no capsule or vascular contact/invasion; vi) no severe comorbidities (cardiopulmonary, hepatic, or renal)
Cao, 2021 (19)	China-Japan Friendship Hospital, China	Retrospective	MWA	34	8/26	43 ± 11	i) Single isthmus PTMC; ii) no LNM/ETE/distant metastasis; iii) follow-up >6 months; iv) no severe cardiopulmonary, hepatic, or renal failure; v) no coagulation disorders or impaired consciousness
Song, 2021 (15)	Chinese PLA General Hospital, China	Retrospective	RFA	115	18/97	44.9 ± 10.4	i) Single isthmus PTMC; ii) patients underwent RFA; iii) no LNM/ETE/distant metastasis; iv) complete follow-up data

PLA, People’s Liberation Army; TA, thermal ablation; PTMC, papillary thyroid microcarcinoma; PTC, papillary thyroid carcinoma; RFA, radiofrequency ablation; MWA, microwave ablation; LNM, lymph node metastasis; ETE, extrathyroidal extension; SD, standard deviation.

### Characteristics of the ablation methods

Table 3 summarizes the technical parameters of ablation in the included studies. The mean ablation time ranged from 91.75 to 240 s. Operator experience was reported as 3–15 years in four studies, except for one study that did not specify this parameter (18). For local anesthesia, four studies used 1% lidocaine (15, 16, 18, 19), while one study used 2% lidocaine (17). All studies employed the moving-shot technique combined with hydrodissection, with three studies using normal saline (15, 18, 19), one study using saline containing 0.0005% epinephrine (16), and another study utilizing a mixture of normal saline, 5% glucose, sodium hyaluronate gel, or a combination thereof (17), with fluid volumes ranging from 38.1 to 51 mL. Regarding ablation margins, two studies required an ablation zone extending ≥2 mm beyond the tumor edge (17, 19), two studies required ≥3 mm (15, 18), and one study reported an ablation-to-tumor volume ratio of 8.49 ± 4.17 (16). Postoperative ablation completeness was evaluated via contrast-enhanced ultrasound (CEUS) in all studies. Detailed ablation parameters are provided in Table 3.

**Table 3:** Characteristics of the ablation methods.

First author, year	Ablation device	Operators experience (y)	Output power (W)/Output energy (J)	Mean ablation time (s)	Lidocaine	Moving shot	Hydrodissection		Minimum distance of ablation	Confirm of completion
							Solution	Fluid volume (mL)		
Wang, 2025 (16)	Celon AG RFA system (Olympus, Japan) with a disposable bipolar electrode (18 G, active tip: 9 mm)	15	5.84 ± 0.49 W/500 ± 190 J	91.75 ± 32.67	1%	Yes	Normal saline containing 0.0005% epinephrine	38.1 ± 8.4	NR	CEUS
Zhou, 2024 (17)	MedSphere radiofrequency therapy instrument (produced by MedSphere International, Inc., USA) with 18 G disposable RFA needles	10	23.36 ± 9.62 W/NR	151.92 ± 134.77	2%	Yes	Normal saline, 5% glucose, sodium hyaluronate gel, or a combination thereof	NR	At least 2 mm	CEUS
Zheng, 2022 (18)	KY2000 MWA system (Kangyou Medical Instruments, China)	NR	20–30 W/5,888 ± 3,632 J	240 ± 132	1%	Yes	Normal saline	38.4 ± 26.2	At least 3 mm	CEUS
Cao, 2021 (19)	Intelligent basic type microwave tumor ablation system (Nanjing ECO, China) or KY-2000 (Kangyou Medical)	3	30 W/NR	110 ± 45	1%	Yes	Normal saline	51 ± 10	At least 2 mm	CEUS
Song, 2021 (15)	Celon AG RFA system (Olympus) with a disposable bipolar RF electrode (18 G)	10	3–6 W/NR	NR	1%	Yes	Normal saline	NR	At least 3 mm	CEUS

W, Watts; J, Joules; G, gauge; RFA, radiofrequency ablation; NR, not reported; CEUS, contrast-enhanced ultrasound.

### Results of ablation

**Figure 2:**
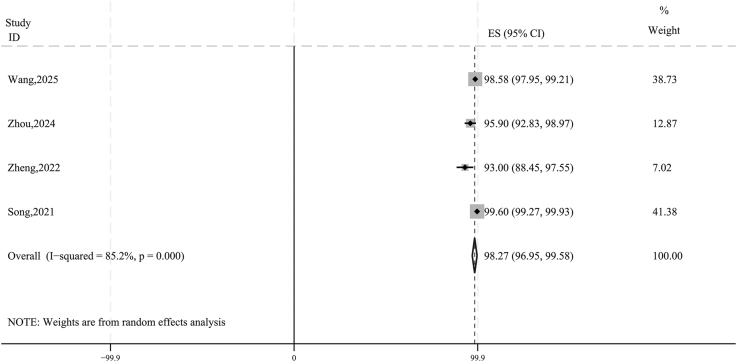
Forest plot: pooled estimates of VRR at the 12-month follow-up.

**Figure 3:**
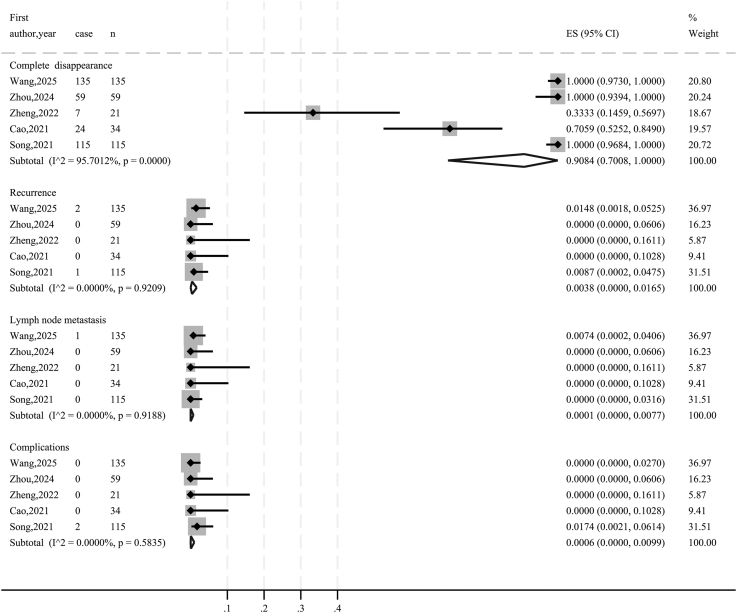
Forest plots: pooled proportions of complete disappearance, recurrence, lymph node metastasis and complications.

As summarized in Table 4, the mean follow-up duration across all studies exceeded 12 months. The pre-ablation tumor size ranged from 5.64 to 6.8 mm in maximum diameter (65.4–181.6 mm^3^ in volume). Excluding one study that did not report VRR (19), the pooled 12-month VRR for the remaining four studies was 98.27% (95% CI: 96.95–99.58; Fig. 2). At the final follow-up, VRR approached 100% in all four studies. A total of 340 tumors achieved complete disappearance, yielding a pooled complete disappearance rate of 90.84% (95% CI: 70.08–100.00; Fig. 3). Local tumor recurrence occurred in three patients (0.38%; 95% CI: 0.00–1.65; Fig. 3), all in the RFA group (15, 16), with no recurrence observed in the MWA group. LNM was reported in one patient (0.01%; 95% CI: 0.00–0.77; Fig. 3).

**Table 4:** Results of ablation.

First author, year	Mean ± SD FU duration (months)	Mean ± SD tumor diameter (mm)/volume (mm^3^)	VRR at the 12-month FU (%)	VRR at the final FU (%)	Complete disappearance	Local recurrence	LNM	Distant metastasis	Complications
Wang, 2025 (16)	24	6.8 ± 1.7	98.58 ± 3.71	100 ± 0	135/135	2/135	1/135	0	None
		136.2 (IQR: 61.2, 174.7)			(100%)	(1.48%)	(0.74%)		
Zhou, 2024 (17)	31.12 ± 12.5	5.64 ± 1.62	95.9 ± 11.78	100 ± 0	59/59	0	0	0	None
		65.4 ± 69.79			(100%)				
Zheng, 2022 (18)	36.1 ± 5.5	6.8 ± 2.0	93 ± 10	100 ± 1	7/21	0	0	0	None
		140.0 ± 120.0			(33.3%)				
Cao, 2021 (19)	17 ± 9	6.0 ± 1.9	NR	NR	24/34	0	0	0	None
		87.4 ± 74.9			(70.6%)				
Song, 2021 (15)	26	6.5 ± 1.9	99.6 ± 1.8	100	115/115	1/115	0	0	Temporary hoarseness: 2 cases (1.7%)
		181.6 ± 156.5			(100%)	(0.9%)			

SD, standard deviation; VRR, volume reduction rate; FU, follow-up; LNM, lymph node metastasis; NR, not reported.

Regarding safety, two patients experienced transient voice hoarseness (0.06%; 95% CI: 0.00–0.99; Fig. 3). Postoperative adverse events occurred in 155 patients (42.58%), including 118 cases of pain (32.42%), six cases of anterior cervical muscle adhesion (1.65%), 26 cases of transient parathyroid hormone elevation (7.14%), one case of cough (0.27%), and four cases of subcutaneous edema (1.10%).

### Quality assessment of the studies

Methodological quality evaluation outcomes are summarized in Table 5. The analyzed studies demonstrated quality scores between 6 and 9 points, reflecting moderate-to-high methodological rigor across all investigations. Two single-arm studies scored 6 points due to limitations inherent in their study design (16, 19). Among the studies scoring 8 points, two exhibited insufficient control of confounding factors, reflecting methodological constraints in addressing potential biases (15, 18).

**Table 5:** Quality assessment of the methodologies of included studies.

First author, year	Selection				Comparability		Outcome		
	Representativeness	Selection of non-exposed	Ascertainment of exposure	Outcome not present at start	Comparability on most important factors	Comparability on other risk factors	Assessment of outcome	Long enough follow-up (median ≥1 year)	Adequacy (completeness) of follow-up
Wang, 2025 (16)	★	-	★	★	-	-	★	★	★
Zhou, 2024 (17)	★	★	★	★	★	★	★	★	★
Zheng, 2022 (18)	★	★	★	★	★	-	★	★	★
Cao, 2021 (19)	★	-	★	★	-	-	★	★	★
Song, 2021 (15)	★	★	★	★	★	-	★	★	★

“★” indicates that study received a score for this item.

## Discussion

PTMC typically exhibits favorable prognosis due to its indolent nature. The 2015 American Thyroid Association (ATA) guidelines recommend AS as the primary strategy for low-risk PTMC (4). A meta-analysis by Cho *et al.* evaluating the long-term AS outcomes reported a 5-year tumor enlargement rate (≥3 mm) of 5.3% (95% CI: 4.4–6.4) and a 5-year LNM rate of 1.6% (95% CI: 1.1–2.4) (20). However, whether AS is applicable to isthmic PTMC remains debated, largely due to the unique anatomical and biological characteristics of isthmic tumors.

Currently, the pathophysiological mechanisms underlying the relationship between the primary site of thyroid tumors and the risk of cervical LNM remain unclear. The lymphatic drainage mechanisms of isthmic PTC are not fully elucidated, and whether isthmic PTC is associated with a high risk of cervical LNM remains inconclusive. Anatomically, the thyroid isthmus has a small volume, thin and narrow shape, with tumors often located close to the thyroid capsule. Due to the close spatial relationship between tumors and the thyroid capsule in isthmic PTC, the incidence of capsular invasion and ETE is significantly higher than in lobar PTC (21). However, the correlation between tumor-to-capsule distance (TCD) and LNM risk remains controversial. Some studies suggest that shorter TCD is associated with a higher risk of cervical LNM (22, 23). Nevertheless, Zhu *et al.* found no significant association between TCD and central LNM rates in low-risk PTMC (24). Notably, Seok *et al.* further demonstrated that central and lateral cervical LNM rates in isthmic PTC were comparable to those in lobar PTC, and ETE was not an independent risk factor for LNM. The prognostic value of ETE in isthmic PTC requires further investigation (25).

In addition, isthmic PTC shows a higher likelihood of multifocality (2), and multifocal lesions have been reported to correlate significantly with the increased rates of capsular invasion, ETE, and LNM (26). However, Wang *et al.* challenged this view, demonstrating that multifocality has no significant impact on recurrence-free survival (RFS) or disease-specific survival (DSS) in PTC patients and recommending against aggressive treatment was solely based on multifocality (27).

Current clinical practice lacks standardized protocols for surgical intervention strategies in isthmic PTC cases. Some studies suggest that total thyroidectomy (TT) is a reasonable approach for isthmic PTC with tumors >10 mm in diameter (28). Others argue that due to the higher aggressiveness of isthmic PTC compared to lobar PTC, TT should be considered regardless of tumor size (29). However, Park *et al.* reported no significant differences in the 10-year RFS (93.1 vs 94.6%) or DSS (100 vs 100%) between patients undergoing TT and isthmusectomy (30). Thyroid isthmusectomy may serve as a simple and feasible treatment option for patients with isthmic PTC. Compared to TT, isthmusectomy can improve patients’ quality of life, reduce postoperative complications, and potentially avoid the need for postoperative thyroid hormone replacement therapy (31).

Given the controversies and overtreatment risks associated with surgery, TA offers a minimally invasive alternative for low-risk isthmic PTMC. Analysis of 1,613 PTC cases managed with radiofrequency ablation (RFA) revealed sustained progression-free survival rates of 98.0% (1-year), 96.7% (3-year), 96.0% (5-year), and 95.7% (8-year). During a mean observation period of 58.5 months, disease progression occurred in 4.3% of patients, with treatment-related complications observed in 2.0% of the cohort, collectively highlighting the durable therapeutic effectiveness of RFA for PTC (32). A meta-analysis of 5-year TA outcomes reported a 98.5% complete disappearance rate and 1.6% complication rate (33). Compared to these results, our systematic review showed a lower complete disappearance rate (90.84%) but significantly reduced complication (0.06%). In addition, the LNM rate in our study (0.01%) was lower than that reported by Cho *et al.* (1.6%) under AS (20). However, these advantages require validation through longer follow-up.

The anatomical constraints of the thyroid isthmus, particularly its proximity to vital structures such as the trachea, have prompted concerns regarding potential thermal damage or incomplete eradication when applying TA to isthmic tumors. These technical challenges have led to cautious approaches in TA utilization for such cases. However, our data revealed no major complications, with only two cases of transient voice hoarseness observed. This favorable safety profile may be attributed to refined operative techniques: i) hydrodissection creates a protective fluid barrier around the tumor to reduce thermal conduction risks (34, 35) and ii) the moving-shot technique dynamically adjusts the ablation needle position to achieve precise targeting (36). In addition, while Wang *et al.* reported 108 patients with capsular invasion (16), Zheng *et al.*’s prospective cohort study demonstrated that TA remains safe and reliable for PTMC with capsular involvement (37). Regarding controversies over multifocal PTMC, a meta-analysis of four studies showed a pooled complete tumor disappearance rate of 92.8% following TA, with no severe complications reported (38). It is worth emphasizing that hydrodissection and moving-shot techniques effectively prevented the major complication of tracheal injury. However, thermal ablation technology still faces multiple challenges in clinical practice. The primary reason is the lack of standardized reference criteria for selecting ablation devices, methods, and energy settings, resulting in high dependence on operator expertise during implementation. If performed improperly, there remains a risk of serious complications.

Notably, Wang *et al.* reported mild anterior cervical muscle adhesion in six patients postoperatively. This complication may stem from the inflammatory response induced by local thermal injury and the specific anatomical location. On one hand, the thyroid isthmus is adjacent to the anterior cervical muscle groups; thermal energy spreading anteriorly during ablation readily affects the muscles. On the other hand, high temperature causes muscle fiber denaturation and collagen coagulation, triggering a sterile inflammatory response. This promotes fibroblast proliferation and secretion of extracellular matrix, ultimately leading to fibrous adhesions. Although some researchers are concerned that anterior cervical muscle adhesion might increase the difficulty of subsequent surgery, the adhesions resolved within 12–18 months postoperatively in all six patients in this group and mild adhesion did not affect the subsequent surgical management (39).

This systematic review also has certain limitations. First, the high heterogeneity in the 12-month VRR and tumor disappearance rates may arise from the exclusive inclusion of patients with isthmic PTMC treated by thermal ablation, where the inherent lack of control groups could amplify bias and heterogeneity. Second, variations in operator experience, inconsistent follow-up durations, and heterogeneity in baseline characteristics may affect the consistency of outcomes. The restricted number of available studies precluded meaningful subgroup analyses to investigate potential sources of heterogeneity. Third, all studies were conducted at single-center institutions in China, which may introduce regional bias, as outcomes could differ across populations and geographic regions. Fourth, the ablation techniques were limited to RFA and MWA, with no data on LA. Finally, the robustness of the current evidence is constrained by the following factors: i) a small total sample size (*n* = 364); ii) short follow-up periods, insufficient to evaluate long-term recurrence risks; and iii) potential institutional bias from single-center designs.

Despite these limitations, this systematic review and meta-analysis indicate that TA demonstrates promising short-term safety and efficacy for isthmic PTMC. However, these conclusions require validation through future large-scale, multicenter prospective studies with extended follow-up durations (>5 years). Such studies should also compare TA with surgical and AS strategies to refine clinical guidelines and optimize therapeutic decision-making.

## Declaration of interest

The authors declare that there is no conflict of interest that could be perceived as prejudicing the impartiality of the work reported.

## Funding

This work did not receive any specific grant from any funding agency in the public, commercial, or not-for-profit sector.
